# For a Better Quality of Beef: The Challenge from Growing Livestock on Limited Grasslands with a Production–Consumption Balance Perspective

**DOI:** 10.3390/foods12173231

**Published:** 2023-08-28

**Authors:** Zhichao Xue, Huimin Yan, Lin Zhen

**Affiliations:** 1 School of International Economics and Management, Beijing Technology and Business University, Beijing 100048, China; zhichao.xue@btbu.edu.cn; 2 Institute of Geographic Resources and Natural Resources Research, Chinese Academy of Sciences, Beijing 100101, China; yanhm@igsnrr.ac.cn; 3 University of Chinese Academy of Sciences, Beijing 100049, China

**Keywords:** food production, food consumption, grassland carrying capacity, Xilingol League

## Abstract

The growing population, the transition dietary towards animal-based products, and the preference for the brand of grass-feeding livestock are bringing increasing pressure on natural grasslands, especially for dry-land areas. The Xilingol League of China is famous for its free-range livestock product, however, overgrazing and herders’ benefits damage are always serious issues for this semi-arid grassland region. This study focuses on the relationship between the supply of natural grassland and the consumption of free-range livestock in the Xilingol League, and this study employed the grassland carrying capacity as the index to judge the sustainability states and its trends of the local grass-feeding system. Satellite data production of net primary production was used for grassland production, statistical livestock data and the consumption model were used for actual forage consumption, and empirical key informant interview data were used to obtain a more comprehensive understanding. The results show that: (1) the natural grassland carrying capacity of the Xilingol League fluctuated, showing improvement from 2000 to 2021; (2) the grassland management needs to be more diversified in different regions with different natural conditions; and (3) while the demand for free-range, high-quality beef is increasing, attention should be paid to the carrying capacity of natural pastures and more consideration should be taken of the benefits of balancing the livelihood of herders, policy strategies, and the customers’ preferences. Potential ways of doing this include employing technologies to improve livestock production, and further exploring and promoting the economic value of the free-range livestock and the geographical indication to get the economic–ecological win-win situation. The research framework and results would be beneficial to reveal the potential threats in pastoral areas and provide support for the optimization of the regional grass-feeding breeding system, especially in middle-income countries.

## 1. Introduction

The feeding system of livestock reveals the safety, naturalness, and healthiness of meat production, as concluded in [1,2]. Natural grasslands, with biodiversity plants and free-range conditions, have superior positive advantages for the quality of beef and can contribute to the animals’ welfare [3,4]. Grazing in wild grasslands could provide beef that is rich in omega-3 fatty acids, meeting the consumer’s satisfaction [2,5,6]. However, with the growing population, the transition dietary towards more animal-based products [7,8], and the preference for grass-feeding livestock brands, there are also increasing pressures on natural grasslands [9,10]. Approximately 49% of global grasslands are experiencing a decline in vegetation coverage, net primary productivity, and multi-functionality to some extent, and overgrazing has been recognized as one of the main causes [11,12]. Overgrazing is the use of pasture lands beyond the limit of their production capacity or an improper use in terms of grazing period and duration [10,13], and can lead to significant changes in biomass [14,15], floristic composition, diversity [16,17], and soil conditions [18,19]. Globalization is bringing changes and global demand in food circulation and is intensifying the pressure on regional arable lands [20,21].

To relieve the degradation, many high-income countries are shifting towards more intensive cattle production systems for meat and dairy production [22,23,24] with an associated reduction in the use of grassland-based livestock systems [25,26]. However, overgrazing is still the most important driver of grassland degradation, especially in the Eurasian Steppe [27,28]. In China, approximately 90% of grassland areas are degraded and the degradation area is increasing by approximately 2 million hectares per year [29,30]. Mongolia has 76.8% land degradation with extremely severe desertification in over 20% of the area, owing to the climatic challenges and human-induced factors of over-grazing and land use changes [31]. In Kyrgyzstan and Tajikistan, overgrazing regions are mainly located in low-elevation areas near the residential areas, and the overgrazing ratio was 88.4% (sheep unit)/hm^2^ in Kyrgyzstan and 127.8% (sheep unit)/hm^2^ in Tajikistan in 2010 [32]. Thus, for sustainable grazing management, precise animal allocation on the grazing area according to herbage availability is essential to optimize grassland utilization and fulfil the livestock’s nutritional requirements to ensure a good quality of meat [33,34].

The carrying capacity is the amount of forage available for grazing animals in a specific pasture or field for a grazing season. The concept of carrying capacity was first applied in the figurative sense to discuss rangeland productivity and cattle grazing on grasslands [35] and has been widely used for the assessment of stocking rate, sustainable use of natural resources, and range management [36,37]. Carrying capacity can be calculated using a variety of techniques and it usually needs to be monitored and adjusted over time to determine the long-term average in practical applications. The Beef Cattle Research Council (BCRC) of Canada has provided online calculation methods for producers by using provincial production guides of field-based sampling [38] to support individual range management. Remote sensing is more often applied to support carrying capacity assessments with the estimation of biomass [39,40]. The above-ground biomass derived from MODIS NPP proved to be an accurate prediction of grassland biomass in different scales and helped to reveal the overgrazing of mountain grasslands [41] and the decrease trend of carry capacity in Europe and southeastern Brazil [37]. The evaluation of carrying capacity of grassland would be the first step to maintain productivity of both animals and forage while encouraging the quality of meat and sustained health of the grassland resources.

The Xilingol grasslands, located in the Xilingol League, Inner Mongolia Autonomous Region, are an important green ecological barrier and green livestock production and output base in northern China. The meat output of the Xilingol League was 259,000 tons in 2020, occupying approximately 14.5% of the whole province, including 139,000 tons of beef and 120,000 tons of mutton [42]. However, because of increases in livestock and overgrazing since the 1980s, land-use intensity has been substantially strengthened, and degradation and desertification occurred most frequently in semi-arid grasslands [43,44,45], causing serious impacts on livestock breeding and the ecological environment. The evaluation of natural pastures’ ability to feed livestock can provide an important basis for reasonable livestock breeding management and can support accurate market supply. 

The aims of this study are to clarify the carrying capacity of natural grassland in the Xilingol League and, additionally, to find possible methods to ensure the quality of beef and balancing the benefits of herders, grasslands, and consumers. This study evaluates Xilingol’s natural pasture livestock breeding system from two perspectives. Firstly, from the perspective of sustainability statutes, this study evaluated the relationship between the forage production capacity of natural grasslands and actual local livestock by calculating the grassland carrying capacity. Secondly, from the perspective of potential sustainability, this study compared the changing trend of forage supply capacity of natural grassland in the region and the changing trend of raised livestock, and assessed the sustainability of natural pasture feeding systems in the long run. In combination with the empirical experiences of local and indigenous people, the results provide support for the optimization of the regional grass-feeding breeding system and the rational allocation of resource space.

## 2. Materials and Methods

### 2.1. Study Area

**Figure 1:**
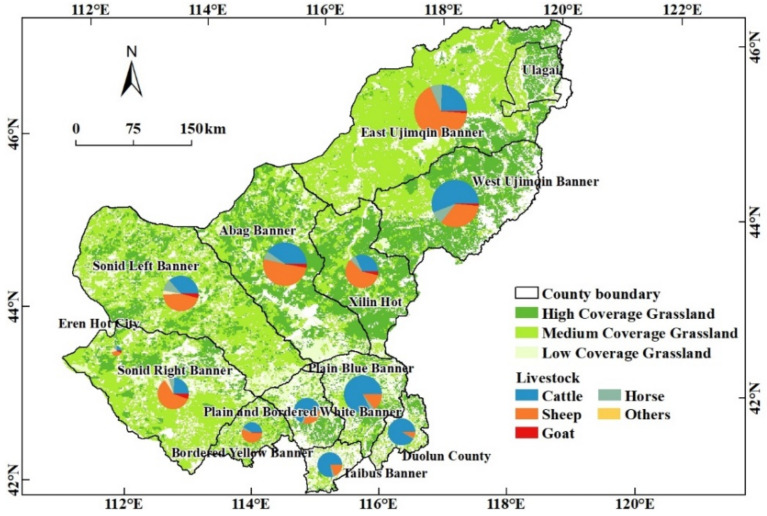
Grassland and livestock in the Xilingol League.

The Xilingol League (115°13′–117°06′ E, 43°02′–44°52′ N) is located in the central part of the Inner Mongolia Autonomous Region. The total land-use area is 2.03 × 10^5^ km^2^, including 1.80 × 10^5^ km^2^ of grassland (Figure 1), accounting for 89.90% of the total area [46]. The regional grassland types mainly include temperate steppe, temperate desert steppe, temperate meadow steppe, and temperate typical steppe. The typical steppe is mainly distributed in the central part of the league, which is the main body of the Xilingol grassland. The terrain is dominated by plains, low mountains, and hills. The usable grassland area of typical steppe accounts for 50.6% of the usable grassland in the league. The surface water in typical steppe is relatively abundant and high-quality forage grass accounts for 50% to 60%. The vegetation is dominated by Stipa baicalensis, Stipa grandis, Stipa krylovii, and Leymus chinense. The Xilingol League is dominated by the semi-arid continental climate, the annual average temperature ranges from 6.54 °C in the southwest to −2.87 °C in the northeast, and the average annual precipitation ranges from 600 mm in the east to approximately 100 mm in the southwest. In 2021, the resident population of the whole league was 1.12 × 10^6^, and the rural resident population was 2.84 × 10^5^. The GDP of the whole league is approximately USD 15.5 billion (1 USD = 6.54 yuan, in 2020), and the proportion of the three major industries is 14.5%, 49.1%, and 36.4%. The per capita disposable income of the whole league is USD 4142.7, and the per capita disposable income of permanent residents in pastoral areas is USD 3175.7. At the end of 2021, the number of cattle raised in the whole league was 1.11 × 10^6^, an increase of 11.1% from the year 2020; the number of sheep raised in the whole league was 5.84 × 10^6^, a decrease of 0.7% compared with 2020. Since 2000, the number of livestock raised at the end of the year in the league showed a trend of decreasing fluctuations. Among them, the amount of large livestock showed a trend of increasing fluctuations, and the amount of sheep feeding showed a trend of decreasing fluctuations [47,48].

In recent years, the Xilingol League has strengthened the protection and utilization of genetic resources for livestock and poultry, and a modern breeding system has gradually formed. The league’s dominant livestock breeds include Sunit sheep, Ujumqin sheep, Chahar sheep, Simmental cattle, Angus cattle, and another 32 breeds, including 9 local breeds, 18 imported breeds, and 5 cultivated breeds. The league has built a national core breeding farm for mutton sheep, 9 breeding sheep farms, 100 multiplication farms, and 1470 core groups. The annual supply capacity of high-quality breeding rams is 35,000. The league also built one national core breeding farm for beef cattle, 22 breeding cattle farms, and 338 core herds, with an annual production of 5000 qualified breeding bulls and 10,000 fine-bred backup cows. At the same time, five local breeds of Wuzhumuqin sheep, Sunit sheep, Mongolian cattle, Mongolian horses, and Abaga black horses have been included in the national livestock and poultry genetic resources protection list. Xilingol mutton has successively won honored awards such as China’s well-known trademark, national geographical indication certification trademark, and the most valuable and influential brand in China’s meat food industry. “Xilinguole Mutton” was selected as one of the top 100 regional public brands of agricultural products in China, and the Xilingol League was recognized as the “Agricultural Products Advantageous Area with Chinese Characteristics” for grassland mutton sheep. In 2021, it was awarded the title of “China’s Ecological Sheep Capital” by the China Meat Association.

### 2.2. Data Source and Research Framework

**Figure 2:**
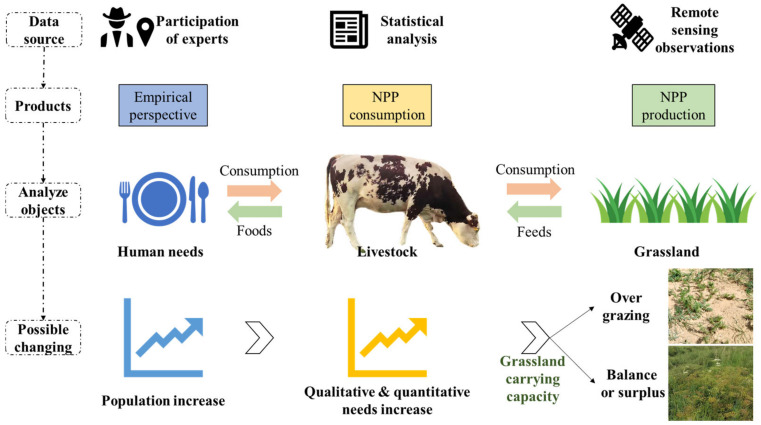
Structure of the designed research.

The data used in this study include spatial distribution of different grasslands, net primary productivity (NPP), statistical yearbook data, and survey data. The spatial distribution of different grasslands was extracted from the land cover and land use data produced by the Resource and Environmental Science Data Center of the Chinese Academy of Sciences (http://www.resdc.cn/, accessed on 8 April 2022). NPP describes the net amount of CO_2_ fixed via plant photosynthesis and is an important indicator for monitoring the growth of vegetation, and is highly sensitive to variations in vegetation productivity [49,50]. The NPP data are based on the MOD09A1 product of Moderate-Resolution Imaging Spectroradiometer (MODIS), combined with the Vegetation Photosynthesis Model (VPM). The spatial resolution is 500 m and the temporal resolution is 8d [51,52,53]. The livestock data of each banner (county), the per capita disposable income of urban residents and pastoral residents, and the total population and labor force of the households in the yearbook are from the 2001-2021 Xilingol League Statistical Yearbook. Livestock data includes the mid-year and year-end numbers of cattle, horses, donkeys, camels, sheep, and goats in each banner (county). We obtained the NPP consumption based on the annual statistical data of livestock. We employed the Grassland Carrying Capacity (GCC) to compare the NPP consumption and production and to further judge the states of grassland pressure. Temporally continuous data were used to diagnose the trends of GCC and to further reflect the sustainability of the local grass-feeding system. The research structure graph can be found in Figure 2 and the detailed calculation process can be found in the following section. 

### 2.3. Production and Consumption State

#### 2.3.1. Natural Grassland Supply

The vegetation photosynthesis model (VPM) is a typical light use model [50,54] that has been widely used and extensively verified for grassland ecosystems’ production estimation [55,56,57]. We selected VPM to simulate the NPP of grassland in the Xilingol League from 2000 to 2019. This research calculates the ratio of natural grassland resource supply of each county to the supply of natural grassland resources in the whole league. The enhanced vegetation index (EVI) and land surface water index (LSWI) were used in the satellite-based vegetation photosynthesis model (VPM) as input data. Both were acquired from the Moderate Resolution Imaging Spectroradiometer reflectance product (MOD09A1 V05; http://ladsweb.nascom.nasa.gov/; accessed on 1 October 2022); 500 m spatial resolution and 8 d time resolution). The EVI adjusts the reflectance in the red band ($\rho_{red}$) as a function of the reflectance in the blue band ($\rho_{blue}$), accounting for residual atmospheric contamination (e.g., aerosols), variable soil, and canopy background reflectance [58]; the LSWI was calculated as the normalized difference between the near infrared band and the short infrared spectral band [59].

#### 2.3.2. Livestock Consumption

The livestock breeding scale of each county is obtained from the year-end livestock inventory in each county. Annual statistics for livestock numbers of the whole Xilingol League, including the numbers of sheep, goats, and large animals were compiled from 2000 to 2019 (except 2012) using the statistical yearbooks of Inner Mongolia [60]. The livestock numbers of each county have not been fully recorded, only the years 2000 and 2013 to 2019 have the numbers of each livestock in the middle of the year and at the end of the year. So, we only analyzed the available years for the counties. To efficiently calculate the grazing intensity, we converted the data for various types of livestock (i.e., sheep, goats, cattle, horses, donkeys, and camels) into standard sheep units based on the conversion coefficient (Table 1). The standard sheep unit conversion coefficient is from the “Calculation of Reasonable Livestock Capacity in Natural Grassland” by the Agricultural Industry Standard of the People’s Republic of China [61] (Table 1). Afterwards, the number of standard sheep units was normalized by the grassland area to obtain livestock densities per unit area.

Based on the number of standard sheep units, the average amount of forage that livestock take and the number of days taken to eat forage, this research estimates the total grassland resource consumption ($CNNP_{i}$) of each county [62,63]:

$$CNNP_{i}=NSSU_{e}\times GW\times GD_{e}\times \left(1-MC\right)\times FC\times 1000+(NSSU_{m}-NSSU_{e})\times GW\times GD_{o}\times \left(1-MC\right)\times FC\times 1000$$

$CNNP_{i}$ represents the total grassland resource consumption (gC), $NSSU_{m}$ and $NSSU_{e}$ represent the standard sheep unit quantity (sheep unit) in the middle of the year (end of June) and at the end of the year (end of December), respectively. GW represents the weight of hay (kg/day), which was set as 1.8 kg/day; $GD_{e}$ and $GD_{o}$ represent the number of days of grazing for livestock remained and livestock sold (day) in the year, which were set as 365 days (184 days in the growing season) and 180 days (61 days in the growing season), respectively. MC represents the water content of air-dried grass, and the water remained on protein and starch in the hay is set as 14% [64]. FC is the conversion coefficient of biomass and carbon content (g C/g). According to the field-measured results of relevant studies, the carbon content of different grass species ranged from 0.45 to 0.55. The value was set as 0.45 in this study [63,65].

#### 2.3.3. Grassland Carrying Capacity 

Grassland Carrying Capacity (GCC) is used as an indicator to indicate the carrying state of grassland resources in a specific year. The calculation formula is as follows:

GCC=CNPP/SNPP

GCC is expressed as the grassland carrying status index ($gC\cdot a^{-1}$), and SNPP is the total amount of grassland supply. When the consumption is lower than the supply, the grass-carrying capacity is in surplus; when the consumption is higher than the supply, the grass is in an overload state. According to the supply of grassland carrying capacity and the degree of surplus [66], carrying status was divided into six levels rich and surplus, surplus, balance surplus, critical overload, overload, and severe overload (Table 2).

#### 2.3.4. Sustainability Trend Analysis

This research calculates the change trends of natural grassland supply in each banner from 2000 to 2019 and compares and analyzes the trend of regional consumption caused by the development of animal husbandry to judge the ability of the region to provide a sustainable resource in the long run. The sustainability trend index of natural grassland feeding systems reflects the security status of grassland resources and the balance of carrying pressure within a period and reveals the potential of regional resource security capabilities and potential threats. Furthermore, the research consisted of interviews of local residents for their perspectives on the development level of green animal husbandry, the utilization efficiency of grassland resources, and the changing trend of biodiversity, as references to assist the explanation of the sustainability trend. Based on the changing trend of forage supply capacity (${Slope}_{SNPPi}$) and the trend of animal husbandry consumption (${Slope}_{CNPPi}$) in each banner, the resource supply and consumption difference (${STI}_{i}$) of grassland in each banner was calculated. If the ${STI}_{i}$ is positive, the regional resource security capability is improving, while the ${STI}_{i}$ is negative, the regional resource security capability is getting worse. The absolute value of the ${STI}_{i}$ represents the degree of change.

$${STI}_{i}={Slope}_{SNPPi}-{Slope}_{CNPPi}$$

Through the descriptive statistical analysis, multi-dimensional diagnosis results of resource carrying status are obtained.

#### 2.3.5. Key Informant Interview

The in-depth interviews of representative herders and local experts were carried out in June, July, and August 2019. To involve different stakeholders and different grassland types of meadow steppe, typical steppe, desert steppe, and different livelihood strategies, the research team selected typical herdsmen and local government officers in the representative counties of East Ujimqin Banner (meadow steppe), West Ujimqin Banner (typical steppe), Xilin Hot City (typical steppe), Plain Blue Banner (agro-pastoral ecotone), Abag Banner, and Sonid Right Banner (desert steppe). The selected person needs to have at least 10 years of living experience in the county and the government officers should be from the relevant grassland and livestock departments [67]. The semi-structured surveys include the basic information of the households’ grassland (or the county’s grasslands for local experts), the perspective of grassland production changing in the past 20 years, the livelihood strategy changing in the past 20 years (e.g., how many sheep and cows did you raise 20 years ago and how has your livestock structure changed in the past 20 years and what are the drivers of these changes?) and the satisfaction of livelihood (e.g., are you satisfied with your living standard? Do you want to have more livestock? Do you want your children to go back to the pastoral area?). A total of 29 valid surveys were conducted. In total, 11 representatives are working in the government department of the Xilingol League, 6 are from West Ujimqin Banner, and 6 are from Sonid Right Banner. There are also 3, 2, and 1 representatives from Plain Blue Banner, East Ujimqin Banner, and Abag Banner, respectively. The information is used to assist the explanation of the results and provide empirical support for a comprehensive understanding and recommendations. 

## 3. Results and Discussion

### 3.1. Supply and Consumption Status: Changes in Grassland Carrying Capacity of the Whole League

**Figure 3:**
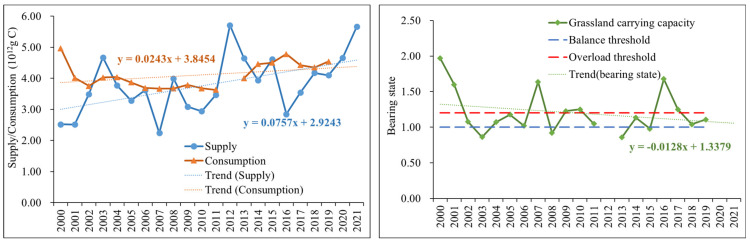
Grassland Supply and Consumption in the Xilingol League. The left is the production of natural grassland and the consumption of livestock from 2000 to 2001; the right is the grassland carrying capacity of grassland in the Xilingol League from 2000 to 2001. (Note: the livestock data of 2012 are missing.).

The supply state of grass production in natural pastures showed a fluctuating rising trend (slope = 0.07) (Figure 3). The large fluctuation is due to the natural grassland, especially the natural grassland in the arid and semi-arid areas, the main factor for the forage production is the influence of climate factors. For instance, 24.01% of the land degradation in Central Asia, which has a typical temperate continental climate zone with sparse vegetation and grasslands accounted for 46.09% and 24.26% of the area, resulted from anthropogenic disturbances [68]. The fluctuation of forage supply in natural pastures is mainly related to the restoration and protection of degraded grasslands measures implemented since 2000 [69,70]. From 2000 to 2010, the six represented national ecological restoration project-induced contribution to C sequestration in China was 770.4 Tg C, representing over half of the total ecosystem C sink for all project regions, the Grassland Conservation project alone has contributed 117.8 Tg C of C sequestration [71]. The consumption of forage grass needed for livestock breeding also fluctuates and rises (slope = 0.02), but the rising trend is not as fast as the increasing trend of natural pasture grass production from the perspective of the whole league. According to the number of livestock raised in the statistical yearbook, since 2000, the number of livestock raised in the Xilingol League has shown a trend of decreasing at the beginning and rising in the following years. In 2000, the number of sheep units in the whole league at the end of the year was 2.10 × 10^7^, and then decreased year-by-year to 1.02 × 10^7^ in 2007, and then showed a fluctuating upward trend, increasing to 1.49 × 10^7^ in 2019.

From 2000 to 2021, the natural grassland carrying capacity of the Xilingol League is fluctuated, decreasing from severe overload to a more balanced state. In 2000, the natural grassland was in the worst state of carrying capacity, and the consumption of livestock breeding was approximately twice the supply of natural grassland. Due to the unrestrained expansion of the livestock breeding scale and overgrazing that gradually increased before 2000, the natural grasslands in the region were obviously damaged, and environmental problems such as sandstorms were caused [72,73]. With the warning of natural disasters caused by unstainable land management, the government has enhanced and launched a series of ecological recovery programs for these sustainable emergencies [74,75], 65% of the total area of China has been included and billions of people are involved in bringing a broad range of land restoration within the next two decades. In the surveyed households, all herders had at least one kind of subsidy, including forbidden grazing, grazing rest, or forage-livestock balance, and had an understanding of managing the livestock size and grazing period to make a sustainable usage of the grassland. In most of the years after 2000 (Figure 3), the carrying state of the natural grassland in the Xilingol League was near to or below the overload level, except for two years 2007 and 2016, which showed serious overload. This is mainly due to the limited natural grass production as shown in Figure 3 (left). 

### 3.2. Supply and Consumption Status: Changes in Grassland Carrying Capacity of Counties

**Figure 4:**
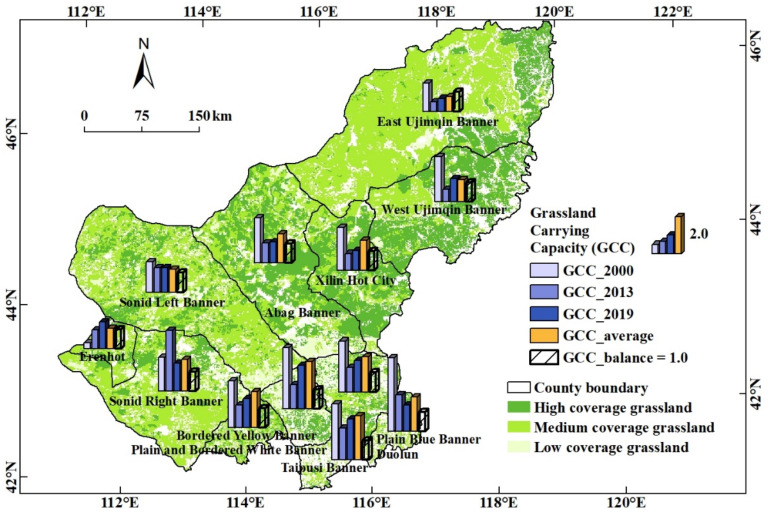
Spatial distribution of grassland carrying capacity in the Xilingol League.

Except for Erenhot, most of the counties in the Xilingol League were seriously overloaded in 2000 (Figure 4). The carrying capacity index of grassland in most counties in 2013 was significantly reduced. Among them, the carrying capacity indexes of four counties were in the range of balance surplus state, and the carrying capacities of two counties were critically overloaded. Compared with the state of carrying capacity in 2000, Sunid Right Banner and Erenhot City increased. Erenhot City was still in a state of surplus balance, while Sunid Right Banner reached a state of critical overload. Sunid Right Banner, which is mainly located in the semi-desert grassland, had an increase in carrying capacity. The carrying capacity of Sunid Right Banner is already exceeded the balance level. The increasing trend shows that the number of livestock breeding is still severely exceeding the level that can be supplied by natural grassland. According to the key informant interview with local government officers in the agricultural department, the local experts addressed the diversity of grassland management in different regions with different natural conditions. The Xilingol League has been empirically divided into the farming-pastoral region, the desert grassland region, and the typical grassland region. The farming-pastoral region is suitable to develop confinement feeding combined with forage planting; desert grassland is recommended to develop confinement feeding to relieve the pressure on natural grassland; and the whole area of the Xilingol League should always be in line with the forage-livestock balance principle. The study concentrated more on the livestock that the natural pasture could support and did not consider the development of silage to assist livestock breeding in the agro-pastoral zone. Regions in the agro-pastoral zone are easy to over-load if they only consider the natural grasslands. Taking full advantage of pasture planting in the agro-pastoral zone and cultivating new breeding varieties to bring better benefits should be a more economically efficient and environmentally friendly method.

From 2013 to 2019, the carrying capacity status index of 10 of the 12 banner counties in the league increased. Only the carrying capacity of East Ujimqin Banner remains surplus. There are three counties whose carrying capacity is critically overloaded, the rest of the counties are already overloaded or severely overloaded. The overloaded counties are mainly located in the semi-desert grassland area in the south, or in the agro-pastoral transition area, indicating that the grazing pressure on the natural grasslands in these areas is still increasing. According to the average carrying capacity status index in the past five years, only East Ujimqin Banner is in a state of surplus balance, and three counties are in a state of critical overload, while the rest of the counties are in a state of overload or severe overload. Based on the analysis of the year series state indexes, it has been found that the grazing pressure on natural pastures in each county was relatively high in 2000. Since then, the grazing pressure has been well controlled, but after the year 2013, the grazing pressure has shown a rising trend. And, except for East Ujimqin Banner, where there is better precipitation mainly with meadow steppe, the natural pastures of the league are still under heavy grazing pressure. As for the Xilingol League, where the meat production is focused on the brand of free-range cattle and sheep on natural pastures, the expansion of livestock raising will not only pose a threat to the use of local natural pastures and the environment, but will also potentially have a negative impact on brand quality assurance.

### 3.3. Supply and Consumption Trends: Sustainability Trend

**Figure 5:**
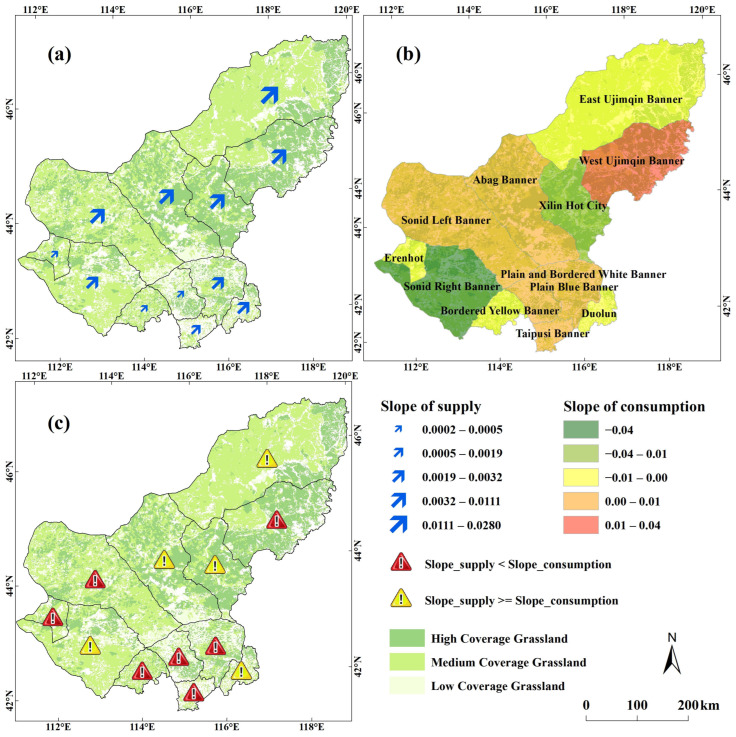
Supply and consumption trends in counties. (**a**) the trend of supply; (**b**) the trend of consumption; (**c**) the comparation of supply trend and consumption trend).

The supply of natural grassland in each county shows a slight upward trend (Figure 5). The greatest upward trend is in East Ujimqin Banner, the slope is approximately 0.03. East Ujimqin Banner is located in the northeast of the Xilingol League and the annual year precipitation is approximately 300 mm, a relatively better condition compared with other areas of the league and the vegetation productivity is more likely to increase as it is dominated by the precipitation [76,77,78]. Livestock consumption is also on the rising trend in most areas of the Xilingol League. Only Xilinhot City, Sunid Right Banner, and Duolun County are in a declining state of consumption, and the most obvious decline trend is in Sunid Right Banner (Slope = 0.05). Sunid Right Banner is located in the semi-desert grassland area, where the reduction of consumption is brought about by the control of livestock quantity, which is conducive to the protection and utilization of natural grassland in the region [70]. West Ujimqin Banner has the most severe consumption increasing trend (Slope = 0.04). West Ujimqin Banner mainly has typical grassland and relevant good grassland coverage conditions. The positive impact of precipitation is likely to cover the negative effect of potential overgrazing, and have adverse impacts in the long run [70]. According to the survey with the expert from the forestry and grassland bureau, the grassland compensation policy played a very positive role in the first five years, and the policy not only brought a good effect on grassland protection, but also promoted the transformation of herdsmen’s perception of reducing livestock breeding. However, in the third five-year round of the policy, herdsmen found that the local livestock did not show positive qualities or a lively mood while they were applying the confinement feeding, so herders gradually turned again to the traditional natural grassland breeding, so the grassland pressure is slightly increased once again. 

Comparing the supply trend change and the consumption trend change in the long-term could provide early warnings for regional sustainability status. There are only five counties in which the increasing speed of supply is faster than that of consumption (Figure 5), and the remaining seven counties are in a state where consumption growth is faster than supply. These trends easily lead to overloading, posing a threat to the regional ecological environment and the quality of grass-based cattle and sheep. There are three types of areas that need a warning with regards to the sustainability trend. One is the semi-desert grassland area in the west, including Sunid Left Banner and Erenhot City. The natural grassland production is insufficient in these areas. If the number and management measures of livestock breeding are not strictly controlled, it will easily lead to regional overloading problems. The second type is the area in the agricultural-pastoral ecotone in the south area, including Taipusi Banner, Plain Blue Banner, Bordered Yellow Banner, and Plain and Bordered White Banner. These areas have a natural condition that is suitable for the development of farming and animal husbandry combined with the breeding mode. The third category is West Ujimqin Banner, which is located in a typical grassland area. In this area, more attention should be paid to the control of the grass and livestock balance and thus potential overload across a long period of time would not be overlooked [70]. In summary, while the demand for free-range, high-quality beef is increasing, attention should be paid to the carrying capacity of natural pastures and to the sustainability threats in pastoral areas with different natural conditions.

### 3.4. From the Perspective of Herdsman: Dependence on Natural Pasture and Ways to Ensure Highly Beneficial Beef Production

#### 3.4.1. Herders’ Dependence on Natural Pastures and Production Improvement Potential 

Livestock production creates a livelihood for one billion poor people through their pastoralist livestock husbandry [27,79]. Approximately 12 million households in China belonging to herdsmen are on the grasslands and they mainly rely on grazing for their livelihood. Livelihood and grassland dynamics are strongly coupled because resource use patterns of local herders have substantial implications for rangeland vegetation, and changes in vegetation in turn shape pastoralist livelihoods [80,81]. Herdsmen living in pastoral areas have intensive social activities that interact with the grassland ecosystem and should be closely adjusted according to the changing grassland [82,83]. However, half of global rangeland areas are projected to experience a decrease in mean biomass under climate change simultaneously, including Sahel, Australia, Mongolia, China, Uzbekistan, and Turkmenistan, where 376 million people support and 174 million ruminant Tropical Livestock Units [84,85]. 

In addition, there are also potential threats from policy strategies to smallholders’ livelihoods, although these policy strategies have the intention to protect vegetation. In China, continuous grassland management and restoration over the past few decades has achieved remarkable results in increasing vegetation coverage and mitigating ecological degradation [86]. According to the statistic report from the National Forestry and Grassland Administration, since the implementation of the Grassland Ecological Protection Subsidy and Reward Policy in 2011, China has invested more than USD 22.9 billion and the comprehensive vegetation coverage of grasslands in the country has increased from 51% in 2011 to 56.1% in 2020, and the fresh grass production reached 1.1 billion tons. However, these grassland restoration and protection projects also bring new challenges for herders’ living strategy [87,88]. The grassland management regulation declares that grassland grazing by livestock is totally forbidden or that only a very small number of livestock can graze in the severely degraded grassland. However, the perceived subsidies of herdsmen was only approximately 13% to 15% of the marketing value of livestock and cannot compensate for the loss brought about by livestock reduction [12]. Precise grazing management optimizing grassland utilization and enabling nutritional requirements and sufficient output should be well employed in the grazing system [25,89,90]. 

Sustainable management can be achieved by improving productivity through genetic up-gradation, timely health care, and balanced feeding. Currently, livestock productivity is much lower in middle-income countries than in high-income countries, while three fourths of the increasing demand of livestock products coming from middle-income countries [91], and, for instance, account for approximately 95% of the feed biomass in Ethiopia [92]. Some potential reasons for the limited production are unimproved genetic stock, inadequate veterinary provision, and a general scarcity of high-quality inputs [93]. For these ‘knowledge-intensive’ technologies, the failures to adopt improvement technologies usually result from systemic constraints and inadequate attention to sociocultural and economic factors [93]. The government should pay more attention to local herders’ training or to assistance in order to improve the awareness and capacity of local herders [79,94,95]. Building a social-ecological system in pastoral areas that realizes the “win-win” of grassland ecological protection and herders’ livelihood improvement is the foundation and focus of Beautiful China Blueprint, and it is also the realization action for the United Nations Sustainable Development Goals (SDGs), especially for terrestrial ecology (SDG15) and for human well-being (SDG3).

#### 3.4.2. Economic–Ecological Benefit Needs the Co-Work of Different Stakeholders

Traditionally, extensive herding measures lead to perspective of herders that the more they breed, the more income they may get. However, raising less intensively and improving the economic benefits per unit of livestock are potential ways to improve the livelihood of herdsmen and reduce the pressure on pastures. Effective management, which focuses on making more benefits by occupying limited natural resources, is the key concern to realizing economic–ecological coordination on the grassland and improve the resilience to the environment [94]. For instance, cattle on farms in New South Wales and Victoria would have additional weight gain from switching to clean water of at least 6.5% and 1.8% per annum, respectively [96]. In Matiguás, investment and training in pasture management resulted in a 30% and 35% increase in milk production during the wet and dry season, respectively [97]. Additionally, development of the value chain for establishing backward and forward linkages is lacking concern [98,99]. 

On the side of the market system, consumers, especially those in the Chinese market, have not yet fully realized the quality and value of free-range livestock meat products and continue to overlook the added value of the production system behind pasture-raised products for organic or conventional practices. Ruminants convert large quantities of poor-quality herbage unsuitable for humans into highly nutritious energy- and protein-dense human food, which contribute to the nutrition system of human beings and further contributes to the sustainable development goals (SDGs) of food safety. This mode leads to quality labels associated with origin or organic farming, especially in the European area, where there are official labels that identify a superior quality based on environmental quality (e.g., organic farming) or on traditional farming (e.g., ‘TSG’ meaning traditional specialty guaranteed) [100]. A survey in Badajoz, Spain, proved that the price-premium for organic beef is over 40%, with organic-fed-on grass beef preferred slightly over fed-on-concentrate [101]. A survey of 262 Swiss summer farms illustrated that smaller farms can compete with larger farms by maximizing the value added to their products, for instance, by production of high-quality cheese and further diversification of products [102,103]. 

However, less attention has been given to the participation of small-scale livestock producers in value chains [104,105]. The low technical capacity of cooperatives and traders also limits the ability to support farmers. In addition, poor coordination in fulfilling agreements with buyers also limits market opportunities for the entire group [104]. In Tanzania, no more than 15% of all small holders have access to market linkages cooperatives or contract farming, and have potentially more rewarding outcomes [106]. There are also outstanding herder households, who improved the economic benefits by making full use of the natural ecosystem services on limited livestock and also protected the grassland ecosystem [107], and they stand out, having great potential and necessity to transfer. This calls for promotional events and brand-building activities, with assistance from the local government and industry, in order to grow the value of grass-fed beef and lamb and its positioning in the market [6,90] and further improve the participation and benefits of herders in the markets.

## 4. Conclusions

By combining the satellite data and empirical field survey data, this study focuses on the relationship between the supply of natural grassland and the consumption of free-range livestock in the Xilingol League to provide further support for the optimization of the regional grass-feeding breeding system. 

From the perspective of sustainability states, the natural grassland carrying capacity of the Xilingol League has fluctuated, decreasing to a balancing state from 2000 to 2021. Grassland management needs to be more diversified in different regions with different natural conditions. From the perspective of potential sustainability trends, while the demand for free-range, high-quality beef is increasing, attention should be paid to the carrying capacity of natural pastures and more consideration for the benefits balancing the livelihood of herders, policy strategies, and the customers’ preferences should be taken into account. 

Currently, livestock productivity is much lower in middle-income countries than in high-income countries, while the demand for livestock products is increasing with three fourths of the demand coming from middle-income countries. Facing a dilemma situation, as in the Xilingol League, small holders in pastoral areas in developing countries need to improve the livestock production by employing technologies of genetic stock, veterinary provision, and high-quality inputs and developing the value chain for establishing both backward and forward linkages should be a matter of concern. The government should pay more attention to the training of local herders or provide assistance to improve their awareness and capacity. Relevant industries need to further explore and promote the geographical indication value of the free-range livestock to improve the economic benefits per unit of livestock to a reasonable level. The economic–ecological win-win situation should be possible only by combining and involving the multi-stakeholders’ efforts. 

This research set the hypothesis that the grazing strategies in the Xilingol League are approximately consistent with free-range grazing, which has been identified in the field survey. However, it is also possible that some of the herders tried to feedlot partially, which has not been considered in this research and might have brought some errors for the carry capacity of natural grassland. Further efforts into portraying the exact development and gap of the genetic stock, veterinary provision, and feeding quality in the developing area must be carried out, and based on these efforts, it is possible to identify the most urgent aspect and road to economic benefit improvement and meat-quality satisfaction in an eco-friendly manner.

## Figures and Tables

**Table 1:** Conversion Coefficient Table of Standard Sheep Unit.

Livestock	Sheep	Goat	Cattle	Horse	Donkey	Mule	Camel
Conversion coefficient	1	0.8	6	5.5	3	5	8.5

**Table 2:** Classification Standard Table for Grassland Carrying Capacity State.

Classification	<0.6	0.6~0.8	0.8~1.0	1.0~1.2	1.2~1.4	>1.4
Grassland Carrying Capacity	rich and surplus	surplus	balance surplus	critical overload	overload	severe overload

## Data Availability

The data used to support the findings of this study can be made available by the first author upon request.
